# Social context is a cue for tic reduction in clinical settings

**DOI:** 10.1007/s00787-025-02818-2

**Published:** 2025-08-05

**Authors:** Brianna C. M. Wellen, Grace Bacon, David Schneck, Emily Wilton, Alison Pryor, IaOom Khang, Kelvin Lim, Kevin J. Black, Erjia Cui, Mark B. Fiecas, Christine A. Conelea

**Affiliations:** 1 Department of Psychiatry, University of Minnesota Medical School, 2025 E River Parkway, Minneapolis, MN 55414 USA; 2 Carleton College, Psychology, Northfield, MN USA; 3 University of Minnesota, Masonic Institute for the Developing Brain, Minneapolis, MN USA; 4 Northeast Ohio Medical University, Rootstown, OH USA; 5 Departments of Psychiatry, Neurology, Radiology and Neuroscience, Washington University in St. Louis, St. Louis, MO USA; 6 School of Public Health, Division of Biostatistics and Health Data Science, University of Minnesota, Minneapolis, MN USA

**Keywords:** Tourette Syndrome, Tics, Symptom Assessment, Behavior Therapy

## Abstract

Assessment and diagnosis of Tourette Syndrome and other tic disorders relies on clinical observation and self-reported history. However, tics are highly susceptible to contextual influences, including clinical interactions. We used video-based observation to quantify the contextual impact of clinician presence on tics and evaluate the potential for these methods to improve tic detection. Youth ages 12–21 (*N* = 39) participated in a clinical trial with video-recorded pre- and post-treatment assessments. Established methods for precision video-based behavioral coding were used to quantify tic frequency and type across assessment contexts (clinician presence and instruction to suppress tics). Participants had significantly more tics when alone and ticcing naturally (mean tics per minute [tpm] = 25.03) and when alone with suppression instructions (mean tpm = 9.48) than in the clinician’s presence (mean tpm = 3.29), all *p*s <.001. Further, mixed model results showed a significant decrease in tpm across treatment when alone ((β = -21.85; 95% CI: [-33.99, -9.70]), and with a clinician (β = -20.31; 95% CI: [-35.08, -5.55]), but significantly greater decrease in the alone context (β = -6.01; 95% CI: [-9.74, -2.29]). Tics occurred less frequently in clinician presence than alone (even when specifically asked to suppress tics alone), suggesting that the social context of clinician presence may facilitate tic suppression that is automatic and/or learned. Additionally, results establish objective video-based measurement as a valuable tool to detect tics and tic change not visible to the clinician.

Tourette Syndrome and other chronic tic disorders (TDs) are characterized by the presence of motor and/or vocal tics [1]. Population estimates of Tourette Syndrome indicate that it affects 0.4 - 1.1% of the population [2], with school-based studies of TDs identifying symptoms in up to 16% of children [3]. Tics wax and wane over time, are often preceded by uncomfortable premonitory urge sensations, can cause significant disability and impairment [4, 5] and although they are not intentional behaviors, they can often be voluntarily suppressed with effort for brief periods [6]. Phenotypic presentations can vary widely in terms of tic severity, topography, and the presence of co-occurring conditions [7]. Group-level comparisons of individuals with and without tics have shown differences in cortico-striatal-thalamo-cortical circuitry structure and function [7, 8] (CSTC), but no TD biomarkers have been identified that can inform individual-level diagnosis, prognosis, or treatment planning [9]. As a result, TD assessment and diagnosis rely on clinical observation and self-reported history.

The Yale Global Tic Severity Scale [10] (YGTSS), a semi-structured interview conducted by a clinician, is the gold-standard measure for assessing tic severity. Clinicians integrate self-report of tics over the past week with direct observation in real time and rate tics across six dimensions (number, frequency, intensity, complexity, interference, and impairment). Given that retrospective self-report of behavior is prone to error and bias [11], and may omit important details due to its timescale, clinician observations are critical for both YGTSS ratings and TD assessment and treatment. However, the clinical context itself may independently impact tic expression. This raises the possibility that in-clinic observations may not generalize across contexts, an issue that threatens measurement validity (the extent to which a measure captures the “true” state of tics).

Situational factors, including activity, location, emotional state, and the presence or absence of others, have long been known to significantly influence tic expression [12, 13], although the effects of specific contexts can differ across individuals [13, 14]. This contextual reactivity is thought to be caused by a complex interplay between biology and reinforcement learning history (i.e., exposure to tic expression or suppression-contingent consequences). Dopaminergic activity in CSTC circuits encodes learning about consequences, enabling an individual to increase access to reward and avoid punishment in similar situations in the future [15]. In TDs, dopaminergic disturbances are thought to over-strengthen the connections between contextual states and motor plans, such that tic expression and suppression can become state-dependent over time [16]. For example, laboratory-based research has demonstrated that neutral cues can develop stimulus control over tic suppression when paired repeatedly with suppression-contingent reward [17]. Importantly, these conditioning processes can occur outside of the conscious awareness of the individual with tics, such that variability in tics across contexts is not necessarily volitional.

Tic variability across clinical contexts specifically has been previously recognized in the literature. In an early clinical trial [18, 19] researchers observed a larger number of tics on video recordings taken when patients were alone vs. with an examiner, and a second study [20] found idiosyncratic differences in tic frequency across videos taken at home vs. the clinic (for 55% of participants, the contexts were the same, while tics were more frequent in clinic for 15% and at home for 30%). Grossen et al. [21] anecdotally observed 19% of patients to have more tics when alone in an observation room as compared to the clinical interview. Other studies have attempted to more broadly assess the impact of social interactions and/or the presence of others. In self-report surveys of people with tics, the majority report tic worsening and increased self-consciousness about tics in social settings [18], as well as frequent attempts to voluntarily suppress tics in daily life [22]. Of note, the context may impact different tics differentially, as some tics may be more socially valenced or elicit stronger reactions from others.

Taken together, the existing literature suggests that tic expression may be impacted by the clinical assessment context. Currently lacking is a rigorous, quantitative assessment of how tic frequency and tic type may differ by clinician presence, topic of the clinical interaction (tic-focused or not), and tic-specific instructions (tic naturally or suppress) at baseline and longitudinally across treatment.

Accordingly, the aims of the current study were as follows: Aim 1: Compare tic expression (frequency and type) at pre-treatment across four contexts (clinical interaction during the YGTSS; clinical interaction during a non-tic specific assessment [MINI]; alone with instructions to tic freely; or alone with instructions to suppress). This aim answers the question: Is tic expression different across contexts at an initial evaluation? Aim 2: Compare change in tic expression from pre- to post-treatment visits across the video-based Tic Suppression Task [23] (TST) and YGTSS interview contexts. This aim answers the question: Does tic expression change over the course of treatment in certain contexts more than others? Aim 3: Examine the relationship between change in TST-measured tic frequency and YGTSS total tic severity scores. This aim answers the question: Can the TST detect treatment change that the YGTSS does not?

## Methods

### Study design

Data were from a randomized controlled trial (CBIT + TMS trial), which was approved by the Institutional Review Board at the University of Minnesota. Consent and assent (as applicable) were obtained from all participants. Participants received Comprehensive Behavioral Intervention for Tics (CBIT) plus randomly assigned transcranial magnetic stimulation to the supplementary motor area (sham, 1 Hz, or theta burst). Participants completed video-recorded clinical assessments before and after the intervention. Treatment sessions occurred over 10 consecutive business days, with an allowable window of 13 business days to complete the sessions. Treatment started within 10 calendar days of the pre-treatment assessment, and the post-treatment assessments occurred within 10 calendar days of the last treatment session. Trial methods and inclusion/exclusion criteria are fully described elsewhere [24].

### Participants

Participants who completed the trial through post-assessment (*N* = 48) were reviewed for inclusion in the current study. Trial inclusion criteria included: age 12–21 years, current TD meeting DSM-5 criteria, ability to engage in treatment (minimum IQ of 70, English speaking), and stable medication status. Exclusion criteria included: medical conditions or medications contraindicated or associated with altered TMS risk profile, inability to undergo MRI, serious mental health conditions (active suicidality, diagnosis of psychosis or cognitive disability, current substance abuse/dependence), and concurrent psychotherapy focused on tics. Participants were included in the current study if video recordings were available of at least the pre-assessment YGTSS interview and TST, and in which the participant was on camera for the majority of the time, resulting in *n* = 39 participants (*n* = 7 no video recordings, *n* = 2 not on camera for majority of recording).

### Measures

All data relevant to the current study were obtained at the pre-treatment or post-treatment assessment time points.

#### Demographics

Demographic information about participant’s age, sex, gender, race, and ethnicity were collected via self- or parent-report form at the pre-treatment assessment.

#### MINI international neuropsychiatric interview [25, 26] (MINI)

The MINI is a reliable and valid brief diagnostic interview that assesses for the most common DSM-5 psychiatric diagnoses. Administration takes approximately 30–60 min. In the current study, parent presence during this interview was determined based on participant and family preference.

#### Yale global tic severity scale [10, 27] (YGTSS)

The YGTSS is a semi-structured clinical interview. Severity ratings for motor and vocal tics are completed separately and then combined to obtain the total tic severity score (range: 0–50). The YGTSS demonstrates good internal consistency, convergent validity, and discriminant validity. Administration takes approximately 15–40 min for first-time administrations and 5–20 min for subsequent administrations. In the current study, parents were present for participants under age 18.

#### Tic suppression task (TST)

Participants were seated in a clinic room alone in front of a computer and GoPro camera recording at least their upper body and face. Participants were fully informed about the nature and purpose of the video recording (i.e., to enable coders to count the number of tics in the video). Instructions were presented verbally and visually on the computer (instructions: https://www.youtube.com/watch?v=7-d_62nqKrk, task: https://youtu.be/Nhu__2pjGXg). The task encompasses two, three-minute conditions: 1) *“free to tic,”* in which participants were instructed to “tic naturally”, and 2) *“tic suppression,”* in which participants were instructed to “try to stop your tics.” Although deception about recording has been used in some prior TST experiments (e.g., [23]), deception was not ethically justified or scientifically necessary in this study.

### Procedures

#### Participant-facing procedures

Participants completed interviews at two time points (pre- and post-intervention) with a trained study independent evaluator (IE) masked to TMS status. The YGTSS was completed at both time points, and the MINI was completed only at pre-treatment. IE interviews were either administered and recorded via Zoom (88%) or in-person (recorded on a GoPro camera), depending on participant needs and COVID-19 guidelines at the time of data collection. Participants all completed the tasks in the same order, with YGTSS and MINI interview happening before the TST (for some participants they occurred on the same day, but the TST could occur up to seven days following the interview).

#### Coding procedures

Coding methods followed established best practices for tic video-based behavioral coding [28], which were used to quantify tics in all conditions (YGTSS, MINI, and TST). The first 15 min of the YGTSS video (or if < 15 min, the entire recording) and randomly chosen 15-min sections of MINI videos were coded. Coders were masked to TST condition instructions and administration timing (pre- or post-treatment) and the full clips were coded.

All videos were coded for tic occurrences using the open-source software Datavyu [29]. Participant-specific tic definitions were generated based on information from the pre-treatment assessment. Codes captured time of tic-onset, tic-type, and whether or not the participant was off-camera. For tic type, motor tics were coded for bodily location (eyes, eyebrows, nose, mouth, head/neck, shoulders, arms, hands/fingers, chest/torso, and/or legs/feet) and presence of copropraxia (yes/no). Vocal tics were coded for speech or nonspeech and presence of coprolalia (yes/no). Coders were trained via an iterative process that included educational materials, observation, and practice videos. Coders were all research assistants in the lab (undergraduates or post-baccalaureate employees) and had to demonstrate strong inter-rater reliability (at least 80% agreement with an experienced coder) on practice videos before they were considered trained. They additionally participated in weekly consensus/review meetings led by licensed clinical psychologists and were closely supervised on all coding procedures.

Approximately 20% of videos in each context (47 videos total) were coded by two independent coders to confirm inter-rater reliability (IRR). Coder data were transformed into three-second bins. Bins containing one or more tics were labeled as “1” and bins with no tics present were labeled as “0”. Agreement was calculated as the percent of bins where both coders were aligned.

#### Analytic plan

Coding data yielded tic frequency and body part in four separate contexts: YGTSS administration with clinician, MINI administration with clinician, TST Free to Tic (TST-FTT), and TST Suppression (TST-SUP), the latter two in a room alone. Three pre-specified analytic aims are illustrated below. Multiple correction procedures were used for post-hoc analyses.

#### Aim 1. Compare tic expression (frequency and type) at pre-treatment across the four contexts

Two linear mixed models (LMM) were fit, one to examine tic frequency (tics per minute [tpm]), and one to examine tic type range (number of distinct body parts in which a tic occurred). Both incorporated a main effect for the context type (e.g. TST-FTT, TST-SUP, YGTSS interview, and MINI interview), a random intercept for individual participant, and adjustment for age (adjustment chosen based on previous literature). These LMMs account for the correlations of measurements within an individual between assessment types. Post-hoc marginal means were calculated to determine pairwise differences in average tpm between each assessment type, using the Tukey method to adjust for multiple pairwise comparisons.

#### Aim 2. Compare change in tic expression from pre- to post-treatment visits across TST and YGTSS interview contexts

Two separate LMMs were fit to compare the change in mean tic frequency and tic type range across treatment for TST-FTT, TST-SUP, and YGTSS interview contexts. The outcomes for these models were the differences in mean tic expression from pre- to post-treatment, and the main effect was the assessment type (TST vs. YGTSS). A random intercept was included for the individual participants. We also adjusted for the TMS randomization arm (theta burst, 1 Hz, or sham) to account for any differences due to the type of TMS administered.

#### Aim 3. Examine the relationship between change in TST-measured tic frequency and change in YGTSS total tic scores

A linear model was fit to determine if the change in overall tic frequency in the TST conditions from pre-treatment to post-treatment was associated with the change in YGTSS total tic scores from pre to post-treatment. The main effect for this linear model was the change in YGTSS score, and the outcome was the change in tic frequency in the TST conditions. This main effect quantifies the extent to which change in YGTSS scores is associated with change in TST tic frequency. We also adjusted for the TMS randomization arm (theta burst, 1 Hz, or sham) to account for any differences due to the type of TMS administered.

## Results

### Inter-rater reliability

Across all videos, the average agreement was 88% (range: 77% to 100%), consistent with established metrics in the field [30]. Additionally, we calculated kappa agreement values. However, since kappa is greatly affected by variability, it tends to be low for videos where participants have few or no tics. Of the 47 double-coded videos, 39 (79%) had kappa values in the adequate range (kappa > 0.4). Of the eight videos where kappa was below 0.4, for 50%, at least one of the coders had coded two or fewer tics.

### Descriptive results

**Fig. 1:**
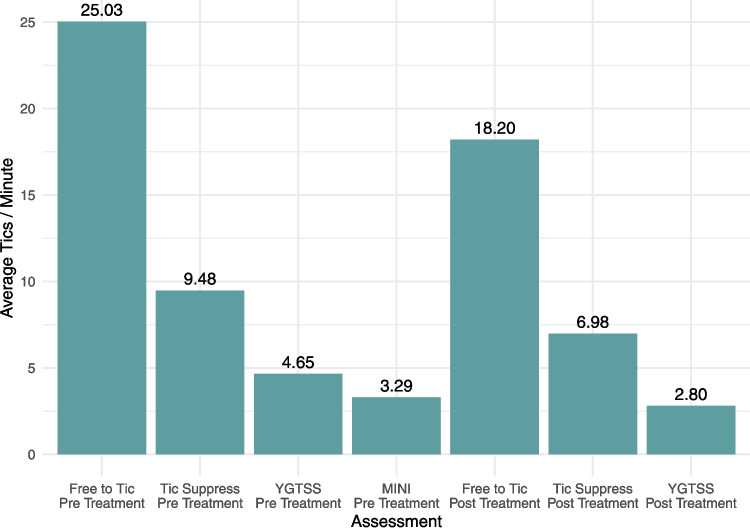
Average tics per minute by condition

Demographic information is represented in Table 1. Average number of tpm per condition are displayed in Fig. 1. For over 95% of the sample, there were more tics in the FTT condition than in the conditions in the presence of the clinician (additional group descriptive statistics outlined in Tables 2 and 3).

**Table 1:** Demographic information

Characteristic	*N* = 39^1^
Age	15.96 (13.40, 18.36)
Sex Assigned at Birth	
Female	17 (44%)
Male	22 (56%)
Gender	
Female	13 (33%)
Male	22 (56%)
Nonbinary	4 (10%)
Ethnicity	
Hispanic or Latinx	4 (10%)
Not Hispanic or Latinx	35 (90%)
Race^2^	
Asian	4 (10%)
Black	1 (2.6%)
Multiracial	3 (7.7%)
White	35 (90%)
Comorbidity	
MDD Current	2 (5.1%)
Anxiety	17 (44%)
PTSD	1 (2.6%)
OCD	6 (15%)
ADHD	15 (38%)
Any Eating Disorder	0 (0%)
ODD (past 6 months)	1 (3.6%)
Conduct Disorder (past 6 months)	0 (0%)
Autism Spectrum Disorder (by history)	2 (5.1%)
CBCL^3^ - Attention Problems	T score = 57 (50,62)
CBCL^3^ - Anxious or Depressed	T score = 54 (50,65)
CBCL^3^ - Obsessive Compulsive Symptoms^4^	M = 0.41, SD = 0.34
Any Psychiatric Meds	21 (54%)
Medication Types	
Vitamin/Supplement	11 (28%)
SSRI	13 (33%)
Alpha-2 adrenergic agonist	10 (26%)
Antihistamine	8 (21%)
Stimulant	7 (18%)
Contraceptive	5 (13%)

^1^Median (IQR); *n* (%), ^2^Participants were instructed to check all that apply. ^3^Child Behavior Checklist, a validated and normed measure, was administered to *n* = 28 who were in the appropriate age range. ^4^The Obsessive Compulsive Symptom Subscale is calculated as an average of the eight relevant items. The score from the current sample is lower than an OCD sample but higher than a psychiatrically treated sample

**Table 2:** Proportion of participants experiencing each tic type

Tic Type	*N* = 39
Eye/eyebrows	39 (100%)
Head/neck	34 (87%)
Mouth	34 (87%)
Nose	32 (82%)
Simple vocal (e.g., sniffing)	29 (74%)
Shoulder	26 (67%)
Arm/hand/fingers	22 (57%)
Chest/stomach/torso	16 (41%)
Legs/feet	6 (15%)
Copropraxia	3 (7.7%)
Vocal tic (speech)	2 (5.1%)
Coprolalia	0 (0%)

If a tic met the definition for copropraxia, it was only coded as copropraxia and not additionally in the respective body part

**Table 3:** Paired condition comparisons for tic frequency

A had more tics than B	Percentage greater (A > B)
Pre Free to Tic > Pre YGTSS	95% (37/39)
Pre Free to Tic > MINI	97% (38/39)
Pre YGTSS > MINI	74% (29/39)
Pre Free to Tic > Pre Suppress	82% (32/39)
Pre Suppress > Pre YGTSS	67% (26/39)
Pre Suppress > MINI	77% (30/39)
Post Free to Tic > Post YGTSS	100% (33/33)
Post Free to Tic > Post Tic Suppress	84% (31/37)
Post Tic Suppress > Post YGTSS	74% (25/34)

*Pre* Pre-treatment. *Post* Post-treatment

### Analysis results

#### Aim 1. Comparisons of context at pre-assessment timepoint

Tic frequency (Fig. 1): Mean tic frequency at pre-treatment was significantly higher during the TST-FTT condition than the TST-SUP condition, YGTSS interview, and MINI interview after adjusting for the age of the participant (all *ps* < 0.001, see Table 4). Post-hoc results: tic frequency was significantly higher in the TST-SUP condition than the MINI interview (mean difference of 5.18 tpm, 95% CI: [0.12, 10.25], *p* = 0.043), but the difference in tic frequency between TST-SUP and YGTSS interview conditions was not significant (mean difference of 4.09 tic/minute, 95% CI: [−1.06, 9.24], *p* = 0.1).

**Table 4:** Aim 1 results

Results (Baseline)	Assessment	β (95% CI)	*p* value
Aim 1: Tic Frequency^A^	TST-FTT		
	TST-SUP	14.01 (10.16, 17.86)	< 0.001
	MINI	19.19 (15.34, 23.04)	< 0.001
	YGTSS	18.10 (14.19, 22.01)	< 0.001
Aim 1: Tic Types^A^	TST-FTT		
	TST-SUP	1.85 (1.08, 2.61)	< 0.001
	MINI	2.01 (1.23, 2.79)	0.011
	YGTSS	1.00 (0.24, 1.76)	< 0.001

A. Results from baseline LMMs adjusted for age where FTT is used as the reference group. The reported β is the mean difference in tic severity between conditions adjusted for age and the correlation arising from repeated within-subject measurement

#### Range of tic types

Similarly, tics occurred in significantly more body parts during the TST-FTT condition compared to the TST-SUP condition, YGTSS interview, and MINI interview (see Table 4). Post-hoc analyses showed that there were no significant differences in the range of body parts in which tics occurred between TST-SUP conditions and either the MINI interview (mean difference of 0.16, 95% CI: [−0.87, 1.19], *p* = 0.98) or the YGTSS interview (mean difference of 0.85, 95% CI: [−0.19, 1.89], *p* = 0.15).

#### Aim 2. Compare change in tic expression from pre- to post-treatment visits across TST and YGTSS interview contexts

For each of the following models, the reported β is the mean difference in tic severity between pre- and post-treatment, adjusted for TMS condition and the correlation arising from repeated measures on participants. Tic frequency: As illustrated in Table 5, tpm decreased significantly over the course of treatment in the TST-FTT condition, the TST-SUP condition, and the YGTSS interview. Mean tpm decreased significantly more from pre- to post-treatment in the TST-FTT context than in the YGTSS interviews. There was no significant difference in the change in mean tic frequency between TST-SUP and YGTSS.

**Table 5:** Aim 2 results

Results (Pre - Post)	Assessment	β (95% CI)	*p* value
Aim 2: Tic Frequency	TST-FTT^A^	−21.85 (−33.99, −9.70)	< 0.001
	TST-SUP^A^	−7.19 (−14.31,−0.07)	0.048
	YGTSS^A^	−20.31 (−35.08, −5.55)	0.008
	FTT vs. YGTSS^B^	−6.01 (−9.74, −2.29)	0.003
	TST vs YGTSS^B^	−1.29 (−3.91, 1.33)	0.30
Aim 2: Tic Types	TST-FTT^A^	−0.57 (−1.17, 0.03)	0.062
	TST-SUP^A^	−0.55 (−1.16, 0.06)	0.074
	YGTSS^A^	−0.97 (−1.70, −0.24)	0.011
	FTT vs. YGTSS^B^	0.380 (−0.56, 1.32)	0.40
	TST vs YGTSS^B^	0.40 (−0.40, 1.20)	0.30

A. Results from LMMs adjusted for age. Each β is the difference in outcomes between pre- post-treatment within an Assessment type

B. Results from linear regression models adjusted for age on the differences between pre-post treatment between Assessments

#### Range of tic types

Tic type range decreased significantly in the YGTSS interview context (Table 5). Although the range of tic types generally decreased over the course of treatment in the TST-FTT condition and the TST-SUP condition as well, these changes were not significant. Mean tic type range decreased more from pre- to post-treatment in the YGTSS interviews than in the TST-FTT condition or the TST-SUP condition.

#### Aim 3. Examine the relationship between change in TST-measured tic frequency and change in YGTSS total tic scores

**Fig. 2:**
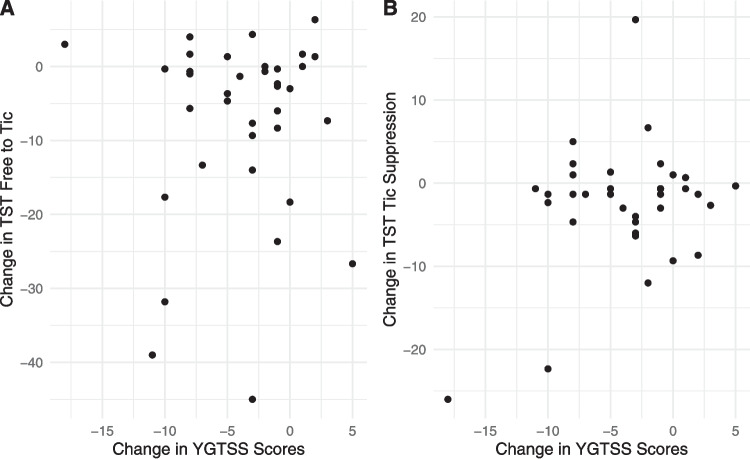
Association between YGTSS score change and TST score change

All analyses adjusted for treatment arm assignment. We observed a significant reduction in clinician-rated YGTSS total tic severity scores from baseline to post-treatment (β = −3.66; 95% CI: [−5.19, −2.13], *p* < 0.001, cohen’s *d* effect size = 0.80). However, there was not a significant relationship between the change in mean tpm in the TST-FTT or the TST-SUP and the change in YGTSS total tic severity scores from pre to post-treatment (Fig. 2).

## Discussion

The current study used rigorous video-based behavioral coding methods to quantitatively examine how aspects of the clinical context impact tics and evaluate implications for current assessment techniques. We found that tic severity was context-dependent and that our method for measuring tics could complement current best practices.

### Social context dependency

Our results demonstrated that almost all youth (95%) had significantly higher tic frequency, and on average tics in significantly more body parts, when they were alone ticcing naturally versus with a clinician, consistent with previous research demonstrating the contextual reactivity of tics [12]. This difference was also clinically meaningful: on average, participants had over twice as many tics when they were alone and ticcing naturally compared to any other condition. A particularly novel finding was related to tic suppression. When participants were alone and attempting to suppress, they had significantly *more* tics than during the MINI interview and a statistically *equivalent* frequency of tics during the YGTSS interview. This finding is somewhat surprising, particularly given that tic-related conversation has been thought to increase tics [31]. Although some individuals with tics report that they suppress intentionally in front of others [18], this is likely not ubiquitous enough to explain our results. Additionally, distraction may account for some symptom reduction during conversation, however, research has found that distraction may not reduce tics as much as intentional suppression [32]. Consistent with reinforcement learning principles [17] it appears more likely that the cumulative experience of consequences related to tic expression and suppression in social settings leads to state-dependent engagement of inhibition, resulting in “social automatic tic suppression” [21].

Contrasting with our results, some studies have found that individuals self-report that tics are worse in the presence of others [12, 18]. A notion that reconciles these seemingly discrepant pieces of evidence is the Heisenberg effect, which contends that the very act of measuring or observing a phenomenon alters it [33]. Social context could elicit the Heisenberg effect such that reported tic worsening could reflect heightened self-awareness of tics, secondary emotions, or cognitive attributions (e.g., stress or worry about how others are reacting to tics) that would not be captured in our behavioral coding of tics. Heightened self-awareness may also be linked to premonitory urges, phenomena not examined during the current study, but relevant for future research.

Importantly, although the large majority of the sample followed the same pattern regarding tic frequency across contexts (Table 3), there may be important individual differences (e.g., moderators or confounders) that may predict differences in the pattern found. Specifically, participant age, the degree to which tics are socially-valenced, participants’ level of social cognition [34], social anxiety, self-consciousness about tics, degree of inhibitory control deficits associated with ADHD, whether they are on medication, or how much tics have been functionally reinforced, could impact how they suppress tics across contexts.

### Implications for measurement

The YGTSS, the primary measure of tic severity in clinical trials, relies on both self-report and clinician observation. Our results demonstrate that the clinician-patient interaction itself imposes contextual control over tics, introducing a potential threat to YGTSS measurement validity. When comparing change across measures from pre- to post-treatment, we found that clinician-rated YGTSS total severity scores decreased significantly (consistent with previous CBIT studies [35, 36] and directly observed tic frequency also decreased significantly, both in contexts where the clinician was present (YGTSS) and not present (TST-FTT condition). Interestingly, tic frequency in the TST-FTT Condition decreased *significantly more* than in the YGTSS context, suggesting that alone contexts may be a more sensitive measure of observed tic frequency change. One surprising result was that the YGTSS total scores and TST-FTT tic frequency did not demonstrate convergent validity in our study, such that changes on the TST were not correlated with changes in the YGTSS. This suggests that the TST may be detecting *different* change than the YGTSS. Although the TST may yield a more objective metric of tic frequency than the YGTSS, it is equally important to consider the participants’ perception of their own tic experience when evaluating treatment outcomes. Tic frequency and tic-related distress/impairment can be orthogonal, and some individuals are not bothered by some or all of their tics. Overall, our results demonstrate that the TST is an important tool that could complement YGTSS in measuring symptom change in clinical trials and potentially lend greater specificity to measurement of tic frequency and tic type/anatomical distribution.

### Clinical implications

These results suggest that tics observed during an interpersonal interaction with a clinician are unlikely to comprehensively represent the patient’s experience across contexts. Clinicians should exercise caution in interpreting symptom presence and severity based solely on these interactions. Comprehensive evaluation of tics should include observation of tics without a clinician or others present (e.g., via video or use of observation rooms). Additionally, it may be helpful for patients to bring in video recordings of tics in other contexts. Integrating direct observation with measurement of self-perception of tics may be helpful for identifying intervention strategies that are most likely to provide relief (e.g., for someone with high emotional distress about tics, cognitive restructuring or peer education may be more helpful than focusing exclusively on tic control strategies).

### Limitations and future directions

Unfortunately, we did not ask participants about their perception of their tics or their premonitory urges across the different contexts, which would begin to elucidate some discrepancies between self-reported severity and observed tic severity. Further, our sample may not be generally representative, given that it was unfortunately limited, especially in its racial representation. Methodologically, the current TST procedures did not include deception about the presence of a video-recording device, which may have impacted the degree to which their tic frequencies matched what they would have if participants were truly alone. Additionally, the current measurement of tics could be further improved by ambulatory use of the TST (to understand how tics are impacted across ecologically valid contexts), and using other technologies, such as electromyography (EMG) sensors or AI-processed videos [37]. Improved measurement of symptoms would also allow for more precise and nuanced evaluation of treatments (e.g., for whom they are working and why). Future studies could also add continuous measures of these constructs to start to disentangle patterns for individual participants and should examine these constructs across longer periods, as we did not have the statistical power to conduct follow-up analyses.

## Data Availability

Limited data are available in the NIH National Data Archive. Additional data will be provided upon request if possible by the corresponding author.

## Declarations

### Trial registration number

NCT04578912, registered October 10th, 2020.
